# MEG Network Differences between Low- and High-Grade Glioma Related to Epilepsy and Cognition

**DOI:** 10.1371/journal.pone.0050122

**Published:** 2012-11-14

**Authors:** Edwin van Dellen, Linda Douw, Arjan Hillebrand, Irene H. M. Ris-Hilgersom, Menno M. Schoonheim, Johannes C. Baayen, Philip C. De Witt Hamer, Demetrios N. Velis, Martin Klein, Jan J. Heimans, Cornelis J. Stam, Jaap C. Reijneveld

**Affiliations:** 1 Department of Neurology, VU University Medical Center, Amsterdam, The Netherlands; 2 Department of Clinical Neurophysiology and MEG Center, VU University Medical Center, Amsterdam, The Netherlands; 3 Department of Radiology, VU University Medical Center, Amsterdam, The Netherlands; 4 Department of Medical Psychology, VU University Medical Center, Amsterdam, The Netherlands; 5 Neurosurgical Center Amsterdam, VU University Medical Center, Amsterdam, The Netherlands; 6 Department of Clinical Neurophysiology and Epilepsy Monitoring Unit, Dutch Epilepsy Clinics Foundation, Heemstede, The Netherlands; National Research & Technology Council, Argentina

## Abstract

**Objective:**

To reveal possible differences in whole brain topology of epileptic glioma patients, being low-grade glioma (LGG) and high-grade glioma (HGG) patients. We studied functional networks in these patients and compared them to those in epilepsy patients with non-glial lesions (NGL) and healthy controls. Finally, we related network characteristics to seizure frequency and cognitive performance within patient groups.

**Methods:**

We constructed functional networks from pre-surgical resting-state magnetoencephalography (MEG) recordings of 13 LGG patients, 12 HGG patients, 10 NGL patients, and 36 healthy controls. Normalized clustering coefficient and average shortest path length as well as modular structure and network synchronizability were computed for each group. Cognitive performance was assessed in a subset of 11 LGG and 10 HGG patients.

**Results:**

LGG patients showed decreased network synchronizability and decreased global integration compared to healthy controls in the theta frequency range (4–8 Hz), similar to NGL patients. HGG patients’ networks did not significantly differ from those in controls. Network characteristics correlated with clinical presentation regarding seizure frequency in LGG patients, and with poorer cognitive performance in both LGG and HGG glioma patients.

**Conclusion:**

Lesion histology partly determines differences in functional networks in glioma patients suffering from epilepsy. We suggest that differences between LGG and HGG patients’ networks are explained by differences in plasticity, guided by the particular lesional growth pattern. Interestingly, decreased synchronizability and decreased global integration in the theta band seem to make LGG and NGL patients more prone to the occurrence of seizures and cognitive decline.

## Introduction

Symptoms in patients with brain tumors and in other lesional epilepsy patients are to some extent correlated with histological characteristics of the lesion. For example, most low-grade glioma (LGG; WHO grade 2) patients suffer from seizures. The faster and more invasively growing high-grade gliomas (HGG; WHO grade 3 and 4) more often lead to focal neurological deficits and symptoms due to raised intracranial pressure [1], [2]. Moreover, patients with cerebral lesions suffer from cognitive deficits, for example in the attention domain, that cannot be explained by local disturbance due to infiltration of the lesion [3].

Cerebral lesions such as brain tumors can lead to global alterations in functional interactions, even between brain regions remote from the tumor [4], [5]. This recent insight may increase our understanding of the symptoms in these patients. Differences in symptom patterns might be explained by specific neural network alterations induced by these lesions, possibly depending on pathological background and growth patterns. The brain can be approached as a complex network of interacting brain regions [6]. Functional networks can be studied using neurophysiological recordings such as magnetoencephalography (MEG). Once functional connections between brain areas have been estimated, the resulting brain network can be characterized by concepts originating from graph theory [6], [7], [8]. Several studies have shown that small-world networks, which combine local segregation with global integration, facilitate optimal (brain) network functioning [6], [9], [10], [11].

Loss of small-world characteristics, particularly in the theta frequency range (4–8 Hz), have been shown to correlate with seizure frequency, duration of disease, and cognitive decline in patients with brain tumors and/or epilepsy [4], [12], [13], [14], [15], [16], [17], [18], [19]. However, at this point the picture is far from complete. Previous studies on functional networks in brain tumor patients were mostly based on MEG recordings obtained after neurosurgical intervention or biopsy, while tumor resection has been described to alter functional connectivity [20]. Moreover the contributions of other factors on these network changes, such as genetic predisposition [21], the duration of epilepsy [15], [16], but also the pathology of the underlying lesion, are largely unknown, let alone their interactions. Other network measures than the small-world characteristics described above may yield additional crucial information related to brain functioning in healthy controls and patients suffering from brain diseases. Synchronizability, defined as the stability of the synchronous state [22], , may be of special interest in lesional epilepsy patients, because a seizure can be seen as a temporary transition to a global synchronized state. Indeed, it has been shown that network synchronizability is dynamically altered during epileptic seizures [23]. Synchronizability is related to the topology of the underlying network, but this interaction is complex [24]. The loss of small-world characteristics in the functional networks of brain tumor patients can therefore not be seen as a direct explanation for the vulnerability for epileptic seizures in these patients. Characterization of synchronizability during interictal MEG may provide additional insights on the relation between epilepsy and altered functional networks. Furthermore, functional modules have been identified in the human brain that change during the aging process [25], [26]. Dynamic changes in modularity are related to learning ability, suggesting that the underlying modular structure determines cognitive performance [27]. It has recently been shown that modularity is altered in patients with absence seizures during interictal MEG recordings [28], but no previous work has studied modular characteristics in relation to brain tumors and lesional epilepsy.

In this paper we investigate functional brain networks in LGG and HGG patients. We compare these patients to healthy controls and epilepsy patients with non-glial lesions (NGL). Since epilepsy burden is a known correlate of altered network topology [15], [16], we only studied glioma patients suffering from epilepsy. We hypothesize that networks differ between LGG and HGG patients. We speculate that plasticity effects are reflected in the networks of patients with relatively slow growing lesions such as LGG, in such a way that their networks are more similar to networks of NGL patients than to those in healthy controls or in patients with rapidly growing lesions such as HGG [29]. We expect that changes are mostly seen in the theta band, as functional connectivity in this frequency range is most constantly described to be altered in brain tumor and epilepsy patients [14], [15], [18], [20]. Finally, we aim to show that a change in synchronizability is related to higher seizure frequency, and that disrupted modular network organization is related to poorer cognitive performance.

## Methods

### Subjects

Patients were referred for MEG recordings as part of presurgical evaluation by the Neurosurgical Center Amsterdam between January 2006 and October 2009. Inclusion criteria were: (i) age 18 years or older, (ii) a radiologically identified cerebral lesion confirmed by neuropathology, (iii) a history of seizures. Exclusion criteria for patients and healthy controls were i) prior neurosurgical treatment and ii) a history of neurological disease (other than the inclusion criteria). MEG recordings were obtained prior to neurosurgical intervention. MEG recordings of healthy control subjects were obtained. We divided the patient group into three subgroups according to the subsequent pathological diagnosis of the lesion after surgery: low-grade glioma (LGG; WHO classification grade II), high-grade glioma (HGG; WHO classification grade III and IV) and non-glioma. Seizure frequency (defined as number of seizures per month) and epilepsy duration (defined as time in months since first seizure) at time of MEG recording were calculated to determine the burden of these factors for every patient.

### Ethics statement

Ethical approval was granted by the VU University Medical Ethics Committee. All data were analysed anonymously. Subjects who underwent MEG recordings for research purposes had given written informed consent before participating. All clinical investigations were conducted according to the Declaration of Helsinki.

### Neuropsychological screening

We preoperatively assessed the Stroop color-word test (attention, executive functioning, mental flexibility, mental processing speed), categoric verbal fluency (executive functioning), and the visual verbal learning test (storage and retrieval of verbal memory) in a subset of patients. Scores were compared to those of a healthy control subject (individually matched for age, sex, and educational level) derived from a normative sample [30]. Educational level was assessed with an 8-point scale scoring system, ranging from not having finished primary education (level 1) to having obtained a university degree (level 8) [31]. Patients’ cognitive performance z-scores were calculated for each neuropsychological test score by comparing each person’s score with the mean and standard deviation of the matched healthy controls. In order to obtain a single score on each subtest, different aspects of each test were averaged after conversion to z-scores.

### Magnetoencephalography (MEG)

MEG recordings were obtained using a 151-channel whole-head MEG system (CTF Systems Inc., Port Coquitlam, BC, Canada). Subjects were seated inside a magnetically shielded room during MEG recordings (Vacuumschmelze GmbH, Hanau, Germany). A third-order software gradient was used, with a recording pass band filter of 0.25–125 Hz. Recordings were made during a no-task, eyes closed resting-state condition with a 625 Hz sampling frequency. The headposition relative to the coordinate system of the helmet was recorded at the beginning and end of each recording by leading small alternating currents through three head position coils attached to the left and right pre-auricular points and the nasion on the patient’s head. Changes up to 0.5 cm during recordings were accepted. Recordings of 136 channels were found suitable for analysis in this study; the other 15 channels malfunctioned in at least one of the MEG recordings. For each subject, five artifact free epochs of 4096 samples (6.554 seconds) were carefully selected by visual analysis [L.D./E.D.] and further analysed with the Brainwave software v0.8.83 [authored by C.S.; available at http://home.kpn.nl/stam7883/brainwave.html]. Artifacts were typically due to (eye) movements, drowsiness or technical issues. The length of the epochs was chosen to be 4096 samples as this has proven to be sufficient to detect clinically relevant differences in functional connectivity in previous studies [5], [15], [16]. MEG registrations were converted to datafiles with a coded filename before epoch selection, so the investigators were blind to the subjects’ diagnosis during this process. The selected epochs were filtered in seven frequency bands: delta (0,5–4 Hz), theta (4–8 Hz), lower alpha (8–10 Hz), upper alpha (10–13 Hz), beta (13–30 Hz), lower gamma (30–45 Hz) and higher gamma (55–80 Hz) [32].

### Functional connectivity

Functional connectivity was calculated by means of the phase lag index (PLI), a measure that is insensitive to the effects of volume conduction (see [33] for a detailed description). The PLI calculates synchronization between time series based on the consistency with which one signal is leading or lagging with respect to another signal. It uses the asymmetry of the distribution of instantaneous phase differences between two signals, since a nonzero phase lag between these signals cannot be explained by volume conduction. The PLI ranges between 0 (no asymmetric phase distribution) and 1 (completely asymmetric phase distribution), and has proven to be a useful measure of functional connectivity in several recent MEG studies in our department [14], [15], [34]. An index of the asymmetry of the phase distribution can be obtained from a time series of phase differences ΔΦ (t_k_), k = 1 … N_s_ in the following way:
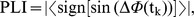
where the phase difference is defined in the interval [-π,π], <> denotes the mean value, N_s_ is the number of samples and t_k_ is the sample index. For each subject, the PLI was calculated between all MEG channels. The overall level of functional connectivity was then computed by averaging all PLI values over all channels. This overall PLI value was used to analyze correlations between the average level of connectivity and lesion pathology.

### Graph analysis

We constructed weighted graphs, in which the edge weight represents the strength of the connection between the vertices. The MEG sensors were considered as vertices (nodes) and the PLI between sensors as edge weights. We calculated the most fundamental network measures, as described by Watts and Strogatz [11], namely the average weighted clustering coefficient C_w_ and average weighted shortest path length L_w_ [34]. The unweighted clustering coefficient describes the likelihood that neighbours of a vertex are also connected, and it quantifies the tendency of network elements to form local clusters. We used the weighted equivalent of this measure to characterize local clustering.

For each vertex i, it is defined as:
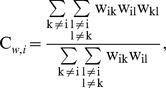
where w_ik_ and w_il_ is the weight between vertex i and vertices k and l, respectively, and w_kl_ is the weight between vertices k and l. The average weighted clustering coefficient is computed by averaging C_w,i_ over all vertices.

The average (weighted) shortest path length indicates the level of global integration of the network. In unweighted networks, it depends on the average number of edges used to connect any two vertices in the network [11]. The average weighted shortest path length (L_w_) is defined as the harmonic mean of shortest paths between all possible vertex pairs in the network, where the shortest path L_ij_ between vertices i and j is defined as the path with the largest total weight [34].
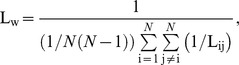
with *N* the number of vertices.

Network properties are determined not only by edge weights and network topology, but also by network size. In order to facilitate comparison of results with other studies, we compared the calculated C_w_ and L_w_ values to a reference, C_ws_ and L_ws_, derived from 1000 surrogate networks of the same size. The surrogate networks were constructed by randomly shuffling the edge weights over the network. The resulting C_w_/C_ws_ and L_w_/L_ws_ are thus the normalized average weighted clustering coefficient and normalized average weighted shortest path length of the network.

Modularity quantifies how a network can be optimally divided in subgroups or modules and was calculated as described by [35], modified for weighted networks by [36]:
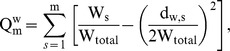
where m is the number of modules, W_s_ is the sum of the weights of all links in the module s, W_total_ is the total sum of all weights in the network, and d_w,s_ is the sum of the weighted degrees of the vertices in module s.

### Simulated annealing

The optimal way to divide the network into modules was then determined using a simulated annealing algorithm [35], [36]. Simulated annealing is an optimization technique that can be used to find an optimal network configuration while considering a cost C. An optimal modularity 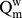, which consists of the largest possible modules, is found for the configuration with the lowest cost C, which is therefore defined as 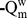. Each of *N* vertices was randomly assigned to one of m possible clusters, where the initial m was taken as the square of N. At each step one of the vertices was randomly chosen and assigned to a different random module number from the interval [1, N]. The new partitioning was preserved with probability:
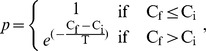
where C_f_ is the final cost and C_i_ is the initial cost, and the temperature T describes to what extent the system allows the exploration of high-cost regions. The temperature T was initially set at 1, and was lowered every 100 steps with T_new_  = 0.995 T_old_. The simulated annealing algorithm ran for 10^6^ steps in total.

### Within-module degree and participation coefficient

We can describe the role of a vertex within a module by calculating its connectivity within that module. The within-module degree (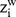) was used to describe to what extend vertex i is connected to other vertices in the same module [37]. A high 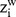 reflects a high within-module degree. The weighted within-module degree is defined as follows:
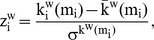
where m_i_ is the module containing node i, 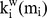 is the within module degree of node i (the sum of all links between node i and all other nodes in module 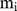, and 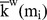 and 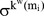 are the respective mean and standard deviation of the within-module degree distribution.

We can also determine to what extend a vertex connects different modules, [37]. The participation coefficient 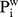 describes how the connections of vertex i are distributed among all modules. The participation coefficient 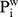 is defined as:
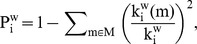
where M is the set of modules, 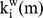 is the sum of all links between node i and all other nodes in module m, and 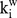 is the sum of all links between i and all other nodes in the network. The 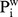 ranges from 0 to 1.

Between-module connectivity P_w_ for the whole network was calculated by averaging all 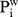, which was used as a measure of connectivity between modules.

### Network synchronizability

We calculated network synchronizability as measured by the eigenvalue ratio R =  λ_N_/λ_2_ to characterize the stability of the synchronous state [22]. For a detailed description we refer to [22], and [38]. In brief, we determined the spectrum of eigenvalues of the graph Laplacian L, which is the difference between the diagonal matrix of vertex degrees and the adjacency matrix. The eigenvalues are then ordered from largest to smallest, being λ_1_  = 0. Networks are more synchronizable when the eigenvalue ratio R is smaller [22]. In order to make results easier to interpret, we define synchronizability S = R^−1^. The synchronizability S is higher for networks with a more stable synchronous state, and S ranges between 0 and 1.

### Statistical analysis

All statistical analyses were performed using PASW 18.0 for Windows (SPSS Inc., Chicago, USA). A one-way ANOVA was performed to test for differences in age between groups. Pearson's Chi square test was performed to test for differences in gender between groups. The PLI and network variables do not follow a normal distribution, hence Kruskal-Wallis tests were performed to explore differences concerning these variables between patients and healthy controls for each frequency band. We corrected for multiple testing using the false discovery rate (FDR) because we performed tests for 5 network characteristics. When a Kruskal-Wallis test showed significant results (p<0.05), post-host analysis was performed by means of Mann-Whitney U tests. Correlations with epilepsy characteristics and cognitive performance were calculated using Kendall's tau tests.

## Results

### Subject characteristics

We included 35 patients (20 male; 13 LGG, 12 HGG, 10 NGL) and 36 healthy controls (18 male). Patient characteristics are shown in table 1. There was a difference in age between groups (F (3,67)  = 6.59; p = 0.001); NGL patients were significantly younger than patients in the other groups. No significant differences in gender were found between groups (Pearson's chi square  = 5.49; p = 0.145). No significant differences regarding epilepsy duration and seizure frequency were found between LGG and HGG patients (Mann-Whitney U = 44.5; p = 0.069 and U = 64.5; p = 0.473, respectively), although epilepsy duration tended to be longer in LGG patients. NGL patients had longer epilepsy duration than LGG (Mann-Whitney U = 12; p<0.001) and HGG patients (Mann-Whitney U = 5; p<0.001). Similarly, NGL patients had higher seizure frequency than LGG (Mann-Whitney U  = 28,5; p = 0.022) and HGG patients (Mann-Whitney U = 17.5; p = 0.004). We found no group differences in the number of anti-epileptic drugs (AEDs) used (Pearson's chi square  = 5.90; p = 0.207).

**Table 1:** Patient characteristics.

Characteristic	LGG	HGG	non-Glioma	Controls
N	13	12	10	36
Age (years)	44.1 (±SD 9.7)	50.3 (±SD 11.5)	30.1 (±SD 6.8)	43.9 (±SD 11.9)
**Gender**				
Male	6	10	4	18
Female	7	2	6	18
**Lesion type**	Grade II: 13	Grade III: 4	DNET: 3	
		Grade IV: 8	MTS: 4	
			HEM: 1	
			HAM: 1	
			DYS: 1	
**Lateralization (lesion)**				
Left	5	3	6	
Right	8	9	4	
**Seizure frequency**	8.2 (±SD 9.9)	17.4 (±SD 43.6)	28.9 (±SD 31.1)	
**Epilepsy duration**	44 (±SD 64)	20 (±SD 39)	228 (±SD 141)	
**Seizure type**				
Part. simple	4	2	1	
Part. complex	0	0	2	
(Sec.) Generalized	9	10	7	
**AED use**				
None	2	0	0	
Single AED	5	9	4	
Multiple AEDs	6	3	6	
**Cognitive performance**	−0.5 (±SD 1.1)	−0.2 (±SD 0.8)		
Attention	−1.1 (±SD 1.8)	−0.5 (±SD 1.1)		
Executive functioning	−1.2 (±SD 1.1)	−1.0 (±SD 0.8)		
Verbal memory	0.0 (±SD 0.9)	0.0 (±SD 0.7)		

Seizure frequency is given per month; Epilepsy history is defined as months passed since first seizure. Cognitive performance scores are presented as z-scores based on individual matched healthy controls. Also, cognitive performance is presented of the domains attention (Stroop test), executive functioning (Verbal Fluency test) and verbal memory (Visual Verbal Learning test). Abbreviations: AED  =  anti-epileptic drug; DNET  =  Dysembryoplastic Neoepithelial Tumor; MTS  =  Mesiotemporal Sclerosis; HEM  =  Hematoma; HAM  =  Hamartoma; DYS  =  Dysplasia.

### Neuropsychological assessment

Cognitive test scores were available for 11 LGG and 10 HGG patients. Cognitive data for NGL patients were available for only 2 patients due to different test paradigms in other patients, and we therefore excluded this group from further analysis. Cognitive performance z-scores based on healthy controls matched for age, gender and educational level are given in table 1. No significant differences in cognitive performance were found between LGG and HGG patients.

### Lesion pathology and functional connectivity

No significant differences were found between any of the patient groups and healthy controls regarding overall PLI level. A non-significant trend was found of higher overall PLI in the theta band in LGG patients compared to HGG patients (Mann Whitney U = 44.5; p = 0.068).

### Lesion pathology and network characteristics

**Figure 1:**
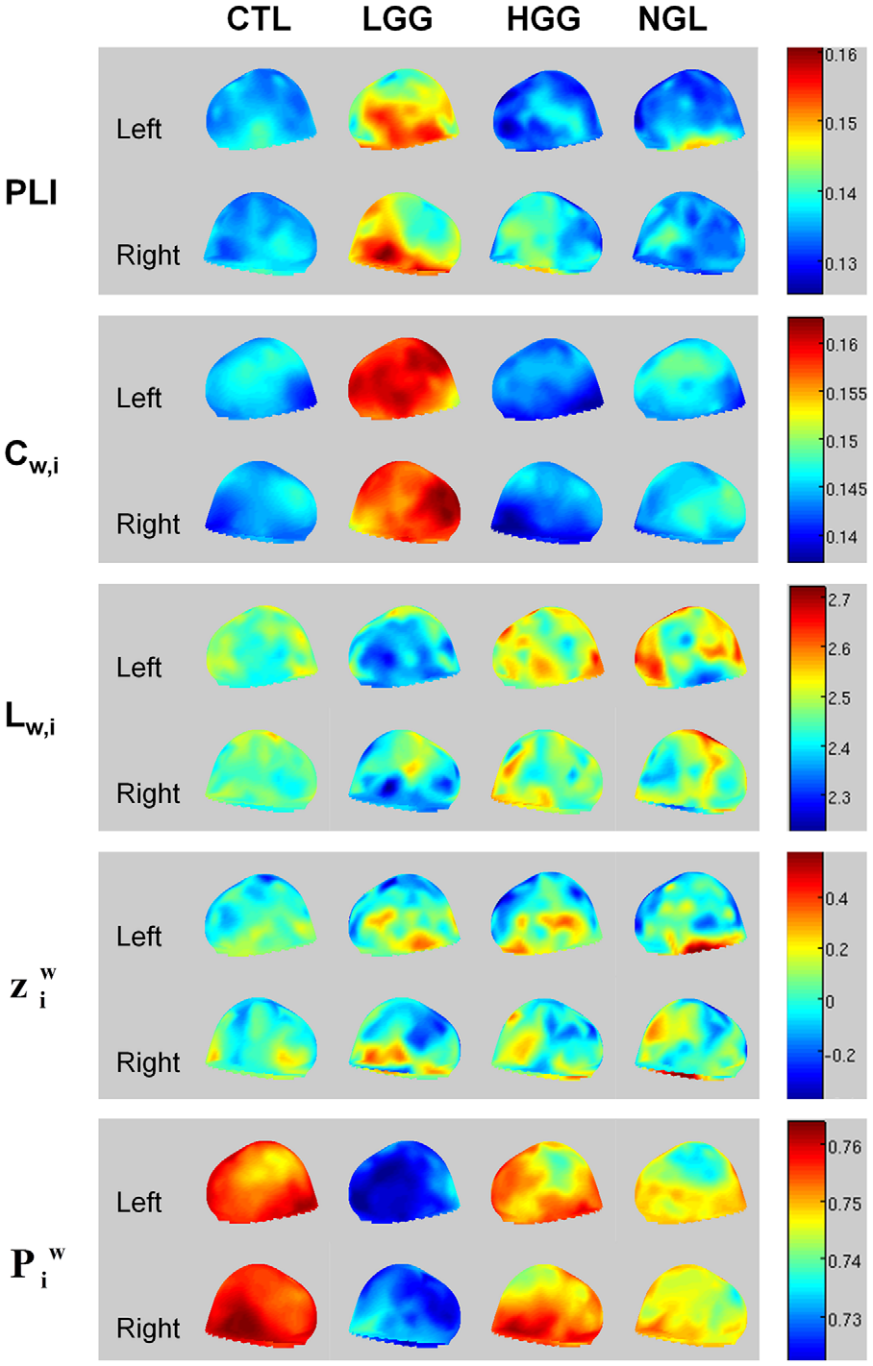
Theta band PLI and network characteristics for patients and healthy controls. Parameters were averaged for each sensor on a group level and displayed on a helmet-shaped surface to show global patterns of differences between patient groups. Note that particularly in LGG patients, theta band clustering and participation coefficients show global alterations irrespective of local PLI values. Abbreviations: CTL  =  healthy controls; LGG  =  low-grade glioma patients; HGG  =  high-grade glioma patients; NGL  =  non-glioma patients; PLI  =  phase lag index; C_w,i_*  =  nodal clustering coefficient; L_w,i_*  =  nodal path length; 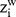  =  within-module degree z-score; 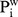  =  participation coefficient. *In the analysis we use normalized average weighted clustering coefficient (C_w_/C_ws_) and normalized average weighted shortest path length (L_w_/L_ws_) instead of the unnormalized values for each vertex i, C_w,i_ and L_w,i_ which are visualized here. C_w_/C_ws_ and L_w_/L_ws_ are calculated by first averaging over nodes and then dividing C_w_ and L_w_ by a reference value C_ws_ and L_ws_, in order to get normalized values. However, this normalization does not affect the spatial distribution of C_w,i_ and L_w,i_, and therefore the original data is presented.

**Figure 2:**
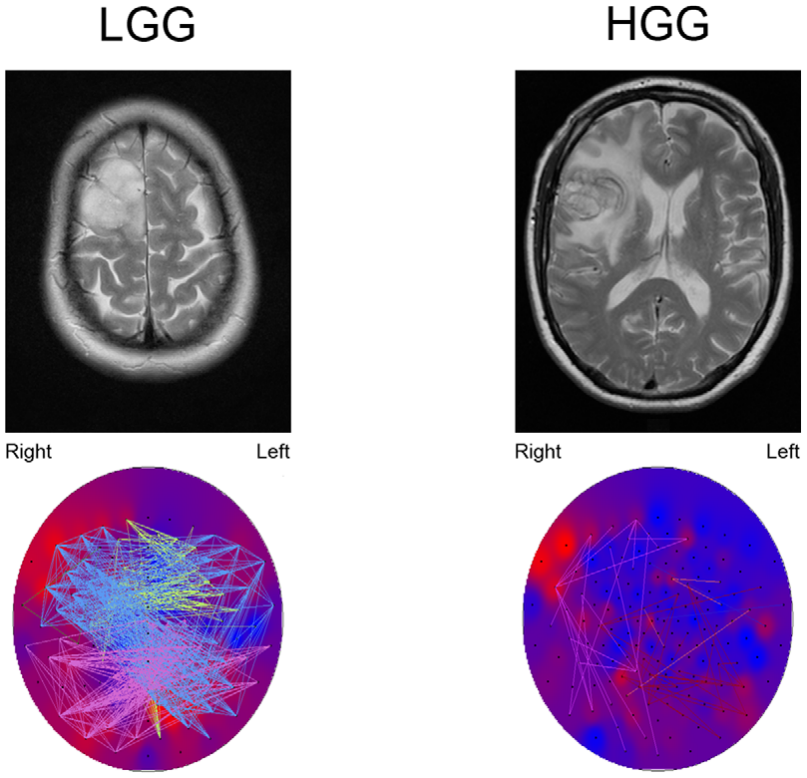
Example of theta band connection differences between a LGG patient and a HGG patient, both suffering from a tumor located in the right frontal lobe. The upper images show T2-weighted MRI images of the tumor. The lower images show theta band PLI levels (background colors; red colors represent high PLI levels, blue colors represent low PLI levels). Note that the tumor region seems to have the highest theta band PLI. The colored lines represent connections between sensors, each color representing another module. Connections are shown when their strength passes an arbitrary threshold chosen for optimal connection visualization. In HGG patients, only few connections exist above the threshold. Note that especially connections to the tumor region in LGG patients pass the threshold. However, two other modules are also clearly shown that are not found in the HGG patient, suggesting that the differences between LGG and HGG patients networks are not restricted to the tumor region.

Kruskal Wallis tests showed that lesion type had a significant effect on normalized weighted clustering coefficient (C_w_/C_ws_), normalized average weighted path length (L_w_/L_ws_), synchronizability (S), modularity (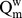) and between-module connectivity (P_w_) in the theta band (Table 2; Figure 1 and 2). Analysis for other frequency bands showed no significant differences between groups. Post-hoc analyses were performed to reveal how the groups differed on these theta band parameters (Table S1). Normalized average weighted clustering was higher in LGG than in healthy controls and HGG patients. Also, LGG patients had lower between-module connectivity than healthy controls, HGG and NGL patients. NGL patients showed higher theta band normalized weighted path length than healthy controls and HGG patients, as well as higher modularity than healthy controls. We found no difference between HGG patients and healthy controls regarding network characteristics.

**Table 2:** Differences between patients and healthy controls regarding theta band network characteristics.

Measure	LGG	HGG	NGL	Controls	p-value
PLI	0.146	0.133	0.136	0.136	0.216
C_w_/C_ws_	1.072 ↑	1.058	1.066	1.058	*0.019
L_w_/L_ws_	1.105	1.084	1.101 ↑	1.087	*0.023
S	0.343 ↓	0.374	0.355 ↓	0.367	*0.009
Q_m_^w^	0.071	0.072	0.075 ↑	0.070	*0.025
P_w_	0.727 ↓	0.750	0.745	0.756	*0.005

Results are given as mean values of network characterstics and p-values of Kruskall-Wallis tests. P-values were considered significant for (p<0.05) after correction using the false discovery rate. Note that the within-module degree z-score (not shown) did not differ significantly.

Results are marked (↑ or ↓) when significantly different from other groups based on post-hoc analyses using Mann-Whitney U tests. Significance levels are given in table S1.

Abbreviations: NGL  =  non-glial lesion; LGG  =  low-grade glioma; HGG  =  high-grade glioma; PLI  =  phase lag index; Cw/Cws  =  normalized average weighted clustering coefficient; L_w_/L_ws_  =  normalized average weighted shortest path length; S  =  synchronizability; 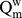  =  modularity; P_w_  =  between-module connectivity.

The number of modules ranged between 5 and 10 for all subjects depending on frequency band, and showed no significant differences between patients and controls (Table S2). Upper alpha band normalized average weighted clustering coefficient (Kendall's tau  =  −0.214; p = 0.009) and normalized weighted shortest path length (Kendall's tau  =  −0.184; p = 0.024) were found to be negatively correlated with age, but we found no significant correlations between age and theta band network characteristics.

As is shown in figure 1, the findings suggest that differences between patient groups regarding network characteristics may be (partly) explained by differences in average PLI levels. We therefore analyzed possible correlations between PLI and theta band C_w_/C_ws_, L_w_/L_ws_, and P_w_ (Table S3). Theta band C_w_/C_ws_ and L_w_/L_ws_ were indeed positively correlated to theta band PLI, whereas a negative correlation was found between P_w_ and theta band PLI.

### Epilepsy, cognition and network characteristics

**Figure 3:**
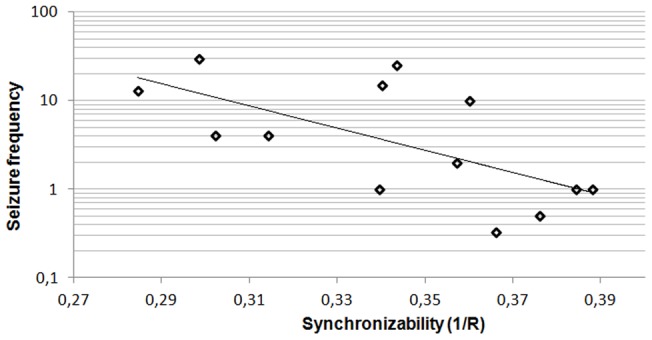
Theta band synchronizability and seizure frequency in low grade glioma patients. Note that seizure frequency is plotted on a logarithmic scale. See tables S4 and S5 for seizure frequency and synchronizability values for each patient.

Post-hoc analysis was performed on network characteristics in the theta band. Higher seizure frequency was associated with lower synchronizability (Kendall's tau  =  −0.448; p = 0.036) in LGG patients, but not in HGG patients (Kendall's tau  = 0.048; p = 0.833) or NGL patients (Kendall's tau  = 0.000; p = 1.000) (Figure 3).

**Figure 4:**
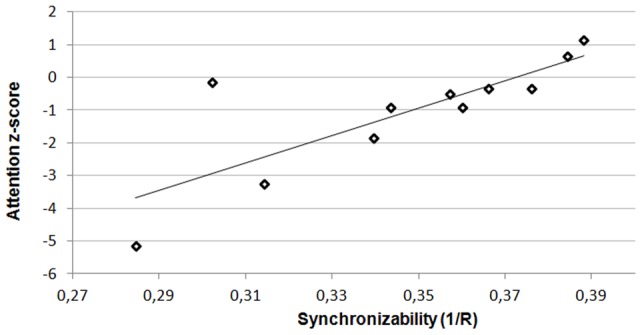
Theta band synchronizability and attention as measured by Stroop tests. Attention scores are presented as z-scores gained by comparison with healthy controls matched for age, gender and educational level. See table S4 for attention scores and synchronizability values for each patient.

Average cognitive test scores correlated positively with theta band synchronizability (Kendall's tau  = 0.661; p = 0.005) in LGG patients, but not in HGG patients (Kendall's tau  = 0.200; p = 0.421). Further analysis showed that in LGG patients, theta band synchronizability correlated positively with attention (Stroop test) and executive functioning (verbal fluency test) (Kendall's tau  = 0.697; p = 0.003 and Kendall's tau  = 0.559; p = 0.020, respectively; Figure 4). Executive functioning was also negatively correlated with normalized average weighted clustering coefficients (Kendall's tau  =  −0.544; p = 0.025), while verbal memory (visual verbal learning test) was positively correlated with modularity (Kendall's tau  = 0.477; p = 0.042) in LGG patients. In HGG patients, we found that higher between-module connectivity correlated positively with better attention test scores (Kendall's tau  = 0.511; p = 0.040).

We found correlations between several theta band network parameters and both cognitive performance and seizure frequency in LGG patients, and it may therefore be that these clinical parameters are also correlated. We calculated the correlation between seizure frequency and cognitive performance in LGG patients and found a non-significant negative trend (Kendall's tau  =  −0.419; p = 0.081).

## Discussion

Our study is the first to show that LGG patients have different neural network characteristics compared to HGG patients (table 3). Functional networks in LGG patients show theta band alterations similar to lesional epilepsy patients with non-glial lesions, while networks in HGG patients are more similar to those in healthy controls. Interestingly, we found topological network differences but no significant differences in general connectivity levels.

**Table 3:** Overview of MEG functional connectivity studies on lesional epilepsy patients.

Study	Population	Methods	Findings
Bartolomei 2006a^1^	17 brain tumour patients vs 15 healthy controls	SL	broad and γ band: disconnected points in brain tumour patients after thresholding SL values
Bartolomei 2006b^1^	17 brain tumour patients vs 15 healthy controls	SL; unweighted networks (k = 10)	Δ, θ and α band: local SL ↑
			Δ, α and β band: long-distance SL ↑
			θ, β, and γ band: L/Ls ↓
			θ, and γ band: C/Cs ↓
Bosma 2008^1^	17 LGG patients vs 17 healthy controls	SL	Δ band: interregional SL ↑or ↓
			θ and lower γ band: interregional SL ↑
			lower α band: interregional SL ↓
Guggisberg 2008^2^	15 focal brain lesion patients vs 14 healthy controls	Imaginary coherence	Decreased α band coherence
Douw 2008^3^	15 brain tumour patients	PLI	θ band: PLI ↓ after resection; higher decrease correlated with lower post-surgery seizure burden
Bosma 2009^1^	17 LGG patients vs 17 healthy controls	PLI; unweighted networks (k = 10)	θ band: PLI and C/Cs ↑
			β band: C/Cs and S ↓
			upper γ band: degree cor. ↓
Horstmann 2009^4^	21 MTLE patients vs 23 healthy controls	cross-correlation; phase sync.; various methods for network construction	broad, Δ, θ and β band: mostly C↑, but also C↓ or = depending on methodology
Douw 2010^1^	17 glioma patients	PLI; weighted networks	θ band: PLI and L/Ls related to higher seizure frequency

Overview of functional connectivity and network studies based on MEG recordings in brain tumour and TLE patients. The measure for functional connectivity used in the study is given in the Methods column. Abbreviations: SL  =  Synchronization Likelihood; PLI  =  Phase Lag Index; L/Ls  =  normalized average path length; C/Cs  =  normalized average clustering coefficient; degree cor.  =  degree correlation (measure for the tendency of vertices to connect to other vertices with a similar degree).

1 MEG recordings used in these studies were obtained after surgery, which might also have had an impact on functional connectivity levels and network topology.

2 No information available on epilepsy incidence in these patients.

3 This study did not compare patients to healthy controls, but compared MEG recordings of patients before and after resection of the brain tumour.

4 This study analyzed patients with non-glial lesions, and should therefore be considered only as a reference for patients with NGL in the present study.

We observed increased normalized theta band path lengths in NGL patients. In contrast, two previous functional MRI studies found smaller normalized average path lengths and lower clustering coefficients in localization-related (non-glioma) epilepsy patients compared to healthy controls [17], [39]. Another MEG study did not find any consistent network differences between NGL patients with epilepsy and healthy controls [40]. As was shown in the current study, differences in lesion pathology between the patient populations in these studies may partially explain these contradictory findings, as well as effects of anti-epileptic drug use and duration of disease [16].

Previous MEG studies comparing functional networks in post-operative glioma patients to those in healthy controls also reported contradicting findings (summarized in table 3). It is important to note here that these studies, especially when reporting on network analysis, were performed after surgical intervention, which has been shown to affect (theta band) connectivity patterns [20]. Patient heterogeneity as well as differences in (network) analysis approaches between these MEG studies and the current study make it even harder to compare results. Some of those previous studies used the synchronization likelihood (SL) as a measure of functional connectivity, which is less conservative than the PLI used in our study, or performed unweighted network analysis. It may thus be that previous studies revealed different aspects of functional network organization in different stages of disease and treatment, rather than being contradictory.

We found the aforementioned differences between LGG and HGG patients in the theta band, while average PLI levels showed a non-significant trend towards higher PLI in LGG patients. The network characteristics were significantly correlated to the overall PLI, even after normalization using random networks of the same size. The possibly higher PLI levels in LGG patients may therefore partly explain the observed differences in network measures. There is currently no optimal method of network construction from functional connectivity matrices that is completely free from biases [41]. The purpose of this study was to find sensitive measures based on functional connectivity between brain areas to differentiate between LGG and HGG patients. We therefore suggest that the network parameters presented here are of additional value compared to the calculation of overall PLI only, and may also provide additional information about the type of connections that are strengthened in LGG patients.

It is hypothesized that plasticity is guided by the particular lesional growth pattern [29]. A recent computational modeling study allowing both growth- and synchronization-dependent plasticity showed that acute lesioning of functional networks leads to increased local clustering levels [36]. Although the model only considered an acute lesion which limits comparability with our study, this is consistent with the increased clustering that we found in LGG patients. However, we found no network differences between HGG patients and healthy controls. A possible explanation is that it might take time before plasticity effects become evident on a global scale, and HGG patients tended to have shorter time between first symptoms and MEG recordings [29]. In the model of Stam and others, however, increased path lengths and decreased modularity were particularly found directly after emergence of the lesion, subsequently normalizing over time [36]. Alternatively, our results may also have been affected by epilepsy characteristics and use of AEDs [15], [16], [17]. Patient groups in our study were relatively small to analyze within group correlations between epilepsy and network characteristics, but we did find a correlation between network synchronizability and seizure frequency in LGG patients. It would be interesting to compare glioma patients with and without epilepsy, and find possible differences in the functional networks of these patients. However, since we found no significant differences between LGG and HGG patients regarding epilepsy duration, seizure frequency and AED use, we consider it unlikely these characteristics would explain differences between these groups.

We found decreased theta band synchronizability, defined as the stability of the synchronous state, in both LGG and NGL patients, and found that lower synchronizability correlated with higher seizure frequency and poorer attention test-scores in LGG patients. Although extremely interesting, these results should be interpreted with caution, as synchronizability was characterized as the stability of the synchronous state, where others use the same terminology to characterize the threshold value of a network for global synchronization [24]. Schindler and others showed that at seizure onset, synchronizability decreases, and increases again at seizure termination [23]. These changes coincided with increased clustering coefficients and path lengths. We suggest that modeling studies on the interaction between network structure and dynamics during seizures are needed to clarify the exact meaning of our observed correlations. The existence of hub nodes with a pathologically increased central role should also be taken into account, as this may be crucial for spreading of epileptic synchronized activity over the network [24], [42], [43], [44], [45]. Future work in which MEG functional networks may be reconstructed in source space is crucial in this respect, which would also allow the identification of anatomical correlates of these pathological hubs, and would increase comparability between subjects [17], [40], [46], [47], [48].

Our findings suggest that in glioma patients a modular brain organization, less local clustering, higher stability of the synchronized state and high between-module connectivity favor cognitive performance. A previous study using post-operative MEG recordings in LGG patients showed that a shorter path length in the delta band was related to better performance in the attention and executive functioning domain, while less local clustering in the lower alpha band was related to better verbal memory test scores, in line with our results [14]. However, another previous study in healthy controls showed an opposite correlation of better attention, working memory and processing speed performance in subjects with higher theta band clustering coefficients [9]. Although that study found correlations with different cognitive domains as compared to our study, and, moreover, healthy subjects instead of brain tumor patients were studied, these findings appear to be contradicting ours. Several other studies have been performed in healthy controls. The most consistent finding seems to be that of a correlation between shorter path lengths and better memory performance or higher intelligence, as this has been established in DTI, MRI and MEG studies [9], [49], [50]. However, an EEG study showed that people with lower education have networks with higher small-world characteristics during a memory task compared to higher educated subjects [51]. This may be interpreted as a reflection of the bigger effort made by subjects with lower education to deliver an equal performance as the subjects with higher education on the task. In general, it could be hypothesized that a small-world topology may be the optimal resting-state organization of healthy brain networks, but that this is not automatically the case for networks in the damaged brain. It could also be that other network characteristics of network topology, such as hierarchical modularity, need to be taken into account in order to capture all the complex interactions between network topology and cognitive performance [52].

The studied domains (attention, executive functioning and verbal memory) specifically require global integration of information. We speculate that modularity and between-module connectivity reflect the facilitation of functional communication. Interestingly, we observed correlations between these network parameters and cognition in the same frequency range, the theta band, as where we observed network differences between LGG patients and healthy controls. The network alterations therefore seem to reflect the less optimal communication within the brain that leads to the impaired cognitive performance in patients with brain lesions. Other cognitive deficits in these patients may also be expected, but no standardized test scores were available in the current study.

We found a non-significant trend towards a negative correlation between epilepsy frequency and cognitive performance. Epilepsy itself can lead to cognitive deficits in brain tumor patients [53]. It might thus be that the network characteristics that we found in these patients are related to either one of these symptoms. Another hypothesis is that the network characteristics may contain information about how recurrent seizures lead to cognitive deficits. The non-parametric distribution of the parameters synchronizability and seizure frequency and the relatively small sample size make the current dataset unsuitable for a regression analysis to clarify these interactions more thoroughly. Also, We corrected for multiple testing per frequency band, as the connectivity matrices provide different information for each frequency band. We performed a Kruskall Wallis test in order to find possible differences regarding any of the metrics, and post-hoc analysis were performed to further interpret results. We suggest that stronger statistical correction would lead to an underestimation of possible group differences and correlations. We note that a correction for multiple testing is not commonly performed for multiple network measures, or average connectivity per frequency band [17], [40], [48].

In conclusion, this study shows that theta band functional networks based on MEG recordings differ in epileptic glioma patients depending on histopathology of the lesion. Lesion type effects are more explicitly seen in LGG and NGL (e.g. MTS) patients when compared to HGG patients, possibly due to plasticity effects that alter brain networks over time. Interestingly, seizure frequency and cognitive decline also correlate with these network alterations. Future studies with larger patient groups should elucidate in more detail the interactions between these clinical characteristics, plasticity and network topology.

## Supporting Information

Table S1 **Network differences between patients and healthy controls.**
(DOC)

Table S2 **Overview of modularity analysis of patient groups and healthy controls.**
(DOC)

Table S3 **Correlations between PLI and several network characteristics in the theta band for all subjects.**
(DOC)

Table S4 **Theta band synchronizability values.**
(DOC)

Table S5 **Values of attention z-scores, seizure frequency (per month) and synchronizability in LGG patients that were used to construct** **figures 3** **and** **4** **.**
(DOC)
