# Supplementary material for: MEG Network Differences between Low- and High-Grade Glioma Related to Epilepsy and Cognition
Source: PLoS One. 2012 Nov 14;7(11):e50122. doi: 10.1371/journal.pone.0050122 (PMC3498183; doi:10.1371/journal.pone.0050122)
Supplement: Table S1 — Network differences between patients and healthy controls. (DOC) [file pone.0050122.s001.doc]

| **Gamma (Cw/Cws)** | |
| --- | --- |
| LGG > Controls | (U = 111.5; p = 0.005) |
| LGG > HGG | (U = 31.5; p = 0.010) |
| **Lambda (Lw/Lws)** | |
| NGL > Controls | (U = 73; p = 0.003) |
| NGL > HGG | (U = 19; p = 0.005) |
| **Synchronizability (S)** |  |
| LGG < Controls | (U = 119.5; p = 0.008) |
| LGG < HGG | (U = 33; p = 0.014) |
| NGL < Controls | (U = 100.5; p = 0.033) |
| NGL < HGG | (U = 24.5; p = 0.018) |
| **Modularity (Q)** | |
| NGL > Controls | (U = 70; p = 0.003) |
| **Between-module connectivity (Pw)** | |
| LGG < Controls | (U = 86; p < 0.001) |
| LGG < HGG | (U = 36; p = 0.022) |
| LGG < NGL | (U = 33; p = 0.049) |

**Table S1** Network differences between patients and healthy controls. Results are given as for each patient group based on Mann-Whitney U tests.
