# Supplementary material for: MEG Network Differences between Low- and High-Grade Glioma Related to Epilepsy and Cognition
Source: PLoS One. 2012 Nov 14;7(11):e50122. doi: 10.1371/journal.pone.0050122 (PMC3498183; doi:10.1371/journal.pone.0050122)
Supplement: Table S2 — Overview of modularity analysis of patient groups and healthy controls. (DOC) [file pone.0050122.s002.doc]

|  |  | **LGG** | | **HGG** | | **non-Glioma** | | **Controls** | | **p-value** |
| --- | --- | --- | --- | --- | --- | --- | --- | --- | --- | --- |
| **Characteristic** | **Freq. range** | Mean | SD | Mean | SD | Mean | SD | Mean | SD |  |
| Modularity (Q) | delta | 0.073 | .0053 | 0.070 | .0061 | 0.075 | .0080 | 0.071 | .0074 | 0.222 |
|  | theta | 0.071 | .0051 | 0.072 | .0049 | 0.075 | .0038 | 0.070 | .0051 | 0.025* |
|  | lower alpha | 0.070 | .0055 | 0.073 | .0076 | 0.075 | .0065 | 0.074 | .0075 | 0.406 |
|  | upper alpha | 0.072 | .0062 | 0.071 | .0073 | 0.076 | .0056 | 0.071 | .0072 | 0.180 |
|  | beta | 0.070 | .0046 | 0.070 | .0074 | 0.070 | .0051 | 0.068 | .0054 | 0.502 |
|  | lower gamma | 0.055 | .0065 | 0.053 | .0048 | 0.056 | .0060 | 0.054 | .0039 | 0.739 |
|  | upper gamma | 0.044 | .0015 | 0.048 | .0061 | 0.047 | .0071 | 0.045 | .0017 | 0.190 |
| No. of modules | delta | 6.4 | .50 | 6.7 | .39 | 6.5 | .48 | 6.7 | .59 | 0.434 |
|  | theta | 6.7 | .46 | 7.1 | .74 | 6.8 | .51 | 7.1 | .48 | 0.143 |
|  | lower alpha | 6.6 | .42 | 6.4 | .87 | 6.7 | .45 | 6.6 | .66 | 0.643 |
|  | upper alpha | 6.7 | .73 | 7.0 | .75 | 6.7 | .67 | 6.8 | .52 | 0.853 |
|  | beta | 7.1 | .58 | 7.0 | .41 | 7.3 | .60 | 7.2 | .62 | 0.561 |
|  | lower gamma | 8.2 | .93 | 8.6 | .75 | 8.2 | .92 | 8.1 | .78 | 0.236 |
|  | upper gamma | 9.8 | .78 | 9.5 | 1.15 | 9.2 | .91 | 9.7 | .59 | 0.265 |

**Table S2**. Overview of modularity analysis of patient groups and healthy controls.
