# Supplementary material for: MEG Network Differences between Low- and High-Grade Glioma Related to Epilepsy and Cognition
Source: PLoS One. 2012 Nov 14;7(11):e50122. doi: 10.1371/journal.pone.0050122 (PMC3498183; doi:10.1371/journal.pone.0050122)
Supplement: Table S3 — Correlations between PLI and several network characteristics in the theta band for all subjects. (DOC) [file pone.0050122.s003.doc]

| **Correlation between PLI and:** | **Kendall's tau** | **p-value** |
| --- | --- | --- |
| Cw/Cws | 0.459 | <0.001 |
| Lw/Lws | 0.341 | <0.001 |
| Pw | -0.252 | 0.002 |

**Table S3**. Correlations between PLI and several network characteristics in the theta band for all subjects (N=71).
