# Supplementary material for: MEG Network Differences between Low- and High-Grade Glioma Related to Epilepsy and Cognition
Source: PLoS One. 2012 Nov 14;7(11):e50122. doi: 10.1371/journal.pone.0050122 (PMC3498183; doi:10.1371/journal.pone.0050122)
Supplement: Table S4 — Theta band synchronizability values. (DOC) [file pone.0050122.s004.doc]

**Table S4.** Theta band synchronizability values.

| **Patient** | **Healthy controls** | **LGG** | **HGG** | **NGL** |
| --- | --- | --- | --- | --- |
| 1 | 0,3998 | 0,3022 | 0,3756 | 0,3722 |
| 2 | 0,3056 | 0,3882 | 0,3652 | 0,3704 |
| 3 | 0,3592 | 0,3436 | 0,3486 | 0,3476 |
| 4 | 0,367 | 0,2846 | 0,3908 | 0,3458 |
| 5 | 0,4058 | 0,3844 | 0,3722 | 0,3384 |
| 6 | 0,339 | 0,3602 | 0,3536 | 0,3374 |
| 7 | 0,392 | 0,3144 | 0,4054 | 0,3466 |
| 8 | 0,412 | 0,3572 | 0,3244 | 0,3520 |
| 9 | 0,3512 | 0,3662 | 0,3876 | 0,3744 |
| 10 | 0,4232 | 0,3396 | 0,3896 | 0,3696 |
| 11 | 0,3838 | 0,3762 | 0,3930 |  |
| 12 | 0,4088 | 0,2986 | 0,3848 |  |
| 13 | 0,3752 | 0,3402 |  |  |
| 14 | 0,369 |  |  |  |
| 15 | 0,3614 |  |  |  |
| 16 | 0,3192 |  |  |  |
| 17 | 0,4202 |  |  |  |
| 18 | 0,3256 |  |  |  |
| 19 | 0,3824 |  |  |  |
| 20 | 0,4062 |  |  |  |
| 21 | 0,3734 |  |  |  |
| 22 | 0,4088 |  |  |  |
| 23 | 0,0722 |  |  |  |
| 24 | 0,3572 |  |  |  |
| 25 | 0,3238 |  |  |  |
| 26 | 0,3414 |  |  |  |
| 27 | 0,3848 |  |  |  |
| 28 | 0,38 |  |  |  |
| 29 | 0,3744 |  |  |  |
| 30 | 0,4078 |  |  |  |
| 31 | 0,3898 |  |  |  |
| 32 | 0,3678 |  |  |  |
| 33 | 0,3678 |  |  |  |
| 34 | 0,3812 |  |  |  |
| 35 | 0,38 |  |  |  |
| 36 | 0,3862 |  |  |  |
