# Supplementary material for: MEG Network Differences between Low- and High-Grade Glioma Related to Epilepsy and Cognition
Source: PLoS One. 2012 Nov 14;7(11):e50122. doi: 10.1371/journal.pone.0050122 (PMC3498183; doi:10.1371/journal.pone.0050122)
Supplement: Table S5 — Values of attention z-scores, seizure frequency (per month) and synchronizability in LGG patients that were used to construct figures 3 and 4 . (DOC) [file pone.0050122.s005.doc]

**Table S5.** Values of attention z-scores, seizure frequency (per month) and synchronizability in LGG patients that were used to construct figures 3 and 4.

| **Patient** | **Attention z-score** | **Seizure frequency** | **Synchronizability** |
| --- | --- | --- | --- |
| 1 | -0.15 | 4 | 0.3022 |
| 2 | 1.13 | 1 | 0.3882 |
| 3 | -0.92 | 25 | 0.3436 |
| 4 | -5.14 | 13 | 0.2846 |
| 5 | 0.65 | 1 | 0.3844 |
| 6 | -0.92 | 10 | 0.3602 |
| 7 | -3.26 | 4 | 0.3144 |
| 8 | -0.50 | 2 | 0.3572 |
| 9 | -0.33 | 0.33 | 0.3662 |
| 10 | -1.84 | 1 | 0.3396 |
| 11 | -0.33 | 0.5 | 0.3762 |
| 12 | N.A. | 30 | 0.2986 |
| 13 | N.A. | 15 | 0.3402 |
